# Bioinformatics and Expression Profiling of the DHHC-CRD S-Acyltransferases Reveal Their Roles in Growth and Stress Response in Woodland Strawberry (*Fragaria vesca*)

**DOI:** 10.3390/plants14010127

**Published:** 2025-01-04

**Authors:** Si Gu, Xinghua Nie, Amal George, Kyle Tyler, Yu Xing, Ling Qin, Baoxiu Qi

**Affiliations:** 1 School of Pharmacy and BioMolecular Sciences, Liverpool John Moores University, Byram Street, Liverpool L3 3AF, UK; s.gu@2020.ljmu.ac.uk (S.G.); a.p.george@2022.ljmu.ac.uk (A.G.); k.d.tyler@ljmu.ac.uk (K.T.); 2 College of Plant Science and Technology, Beijing University of Agriculture, Beijing 102208, China; niexinghuabua@163.com (X.N.); xingyu@bua.edu.cn (Y.X.); qinlingbac@126.com (L.Q.)

**Keywords:** woodland strawberry (*Fragaria vesca*), DHHC-CRD, FvPATs, S-acylation, ABA, SA

## Abstract

Protein S-acyl transferases (PATs) are a family of enzymes that catalyze protein S-acylation, a post-translational lipid modification involved in protein membrane targeting, trafficking, stability, and protein–protein interaction. S-acylation plays important roles in plant growth, development, and stress responses. Here, we report the genome-wide analysis of the *PAT* family genes in the woodland strawberry (*Fragaria vesca*), a model plant for studying the economically important Rosaceae family. In total, 21 ‘Asp-His-His-Cys’ Cys Rich Domain (DHHC-CRD)-containing sequences were identified, named here as FvPAT1-21. Expression profiling by reverse transcription quantitative PCR (RT-qPCR) showed that all the 21 *FvPATs* were expressed ubiquitously in seedlings and different tissues from adult plants, with notably high levels present in vegetative tissues and young fruits. Treating seedlings with hormones indole-3-acetic acid (IAA), abscisic acid (ABA), and salicylic acid (SA) rapidly increased the transcription of most *FvPATs*. A complementation assay in yeast PAT mutant *akr1* and auto-S-acylation assay of one FvPAT (FvPAT19) confirmed its enzyme activity where the Cys in the DHHC motif was required. An AlphaFold prediction of the DHHC and the mutated DHHC155S of FvPAT19 provided further proof of the importance of C155 in fatty acid binding. Together, our data clearly demonstrated that S-acylation catalyzed by FvPATs plays important roles in growth, development, and stress signaling in strawberries. These preliminary results could contribute to further research to understand S-acylation in strawberries and plants in general.

## 1. Introduction

S-acylation, N-myristoylation, and prenylation are lipid modifications of proteins commonly occurring in cells in which a fatty acid is attached to specific amino acid residues, leading to increased hydrophobicity [1]. S-acylation, also known as S-palmitoylation, occurs post-translationally where saturated fatty acid, usually the 16-carbon palmitate, covalently attaches to specific cysteine residue(s) throughout the protein via a thioester bond [2,3]. Both peripheral membrane and transmembrane proteins can be S-acylated, allowing tight association with membranes or membrane microdomains such as lipid rafts, as well as trafficking, regulation, and signaling in the cell [4,5].

A family of protein S-acyl transferases (PATs) catalyze protein S-acylation in the cell. The first identified PAT in 2002, AKR1p, was one of the seven such proteins found in yeast (*Saccharomyces cerevisiae*) [6]. Since then, the structure and functions of this family of enzymes have been reported in various organisms, including plants. Having 4–6 transmembrane domains (TMD), PATs are integral membrane proteins with typical cytosolic N- and C-termini. Importantly, PATs possess a highly conserved DHHC-CRD of ~50 amino acids with a structure of Cx2Cx9HCx2Cx4DHHCx5Cx4Nx3F, usually residing on the cytoplasmic face of the membrane between TMD 2 and 3 [7,8]. The DHHC-CRD domain is the enzyme catalytic center of PATs where the cysteine residue of DHHC attaches a fatty acid to form an acyl intermediate, in the so-called auto-acylation process. This acyl chain is then transferred to the specific cysteine of the target substrate protein to aid its membrane association [6].

To date, 24 AtPATs have been identified in Arabidopsis, where all have 4 TMDs except AtPAT17 with 6 TMDs [9]. AtPAT24 (TIP1) was the first PAT identified in Arabidopsis in 2005 [9]. It is confirmed to be an S-acyl transferase as it can rescue the yeast PAT AKR1 knockout mutant *akr1* for its morphological and temperature sensitive defects. The loss-of-function mutant *tip1* (*atpat24*) exhibited defects in cell size control, cell polarity, pollen tube, and root hair growth [9]. The second plant PAT, AtPAT10, again from Arabidopsis was characterized by us in 2013, 8 years later [10]. The T-DNA knockout transcription null mutant plants of AtPAT10 are extremely dwarfed with smaller and a reduced number of cells. These plants are also near-sterile with very few viable seeds produced. Since then, research in this field has accelerated and a number of PATs have been identified from Arabidopsis (AtPAT4, 13, 14, 15 and 21) [11,12,13,14,15] as well as from crop plants such as rice [16], maize [17], apple [18], and pear [19].

The strawberry is a model plant for the Rosaceae family, which includes many economically important edible fruits, such as apple, pear, peach, and cherry as well as some popular ornamental trees and shrubs, such as roses. To understand the important functions of PATs in strawberries, we searched the recently sequenced genome of the woodland strawberry (*Fragaria vesca*) and found 21 DHHC-CRD-containing sequences, named here as FvPAT1-21. Bioinformatic analysis and expression patterns of all these 21 FvPATs under normal growth conditions as well as under IAA, ABA, and SA treatments were carried out. The verification of the enzyme activity of one FvPAT, FvPAT19, the homolog of Arabidopsis AtPAT14 was conducted by yeast complementation and biochemical assay. The structure of its DHHC and DHHC155S was also predicted by AlphaFold. Data gathered from this study will guide further research into our understanding of PATs in this model plant, providing valuable insights into this family of proteins to guide the molecular breeding of the economically important Rosaceae family of plants.

## 2. Materials and Methods

### 2.1. Identification of FvPATs from Woodland Strawberry Genome

The chromosome-level genome sequences of the updated annotations of Arabidopsis (*Arabidopsis thaliana)* and woodland strawberry (*Fragaria vesca,* ‘Hawaii 4’) were downloaded from the Ensembl Plants datasets (https://plants.ensembl.org/index.html, accessed on 27 April 2024) and Rosaceae Genome database (https://www.rosaceae.org, accessed on 27 April 2024), respectively. To identify protein S-acyl transferases in strawberries, the Hidden Markov Model (HMM) protein signature (PF01529) of the conserved DHHC domain, the characteristic motif of this family of proteins, was downloaded from InterPro (https://www.ebi.ac.uk/interpro, accessed on 27 April 2024) and used to screen FvPATs in the strawberry genome with an E-value of 1 × 10^−10^. The isolated Arabidopsis AtPATs protein sequences [20] were regarded as templates to compare FvPATs with an E-value of 1 × 10^−5^ using the Blastp tool, so that they can be further confirmed individually as homologs of AtPATs. The conserved DHHC domain was further defined among these candidates FvPAT protein sequences via SMART (http://smart.embl-heidelberg.de/, accessed on 27 April 2024) and NCBI (https://www.ncbi.nlm.nih.gov/cdd/, accessed on 27 April 2024). The molecular weight (MW), isoelectric point (pI), and average hydrophobicity of FvPATs were analyzed using TBtools-II while the TMs were predicted by TMHMM (https://dtu.biolib.com/DeepTMHMM, accessed on 27 April 2024).

### 2.2. Multiple Sequence Alignment and Phylogenetic Tree Construction

The protein sequences of the 21 identified FvPATs were aligned with the 24 Arabidopsis PAT sequences using Clustal W in MEGA7.0 [21], to construct a maximum-likelihood (ML) phylogenetic tree based on the WAG substitution model and visualized by iTOL (https://itol.embl.de/, accessed on 27 April 2024). The online OmicStudio platform (www.omicstudio.cn/, accessed on 27 April 2024) was used to analyze and visualize the conserved domains. The conserved amino acids within the DHHC-CRD were created using WebLogo (https://weblogo.berkeley.edu/, accessed on 27 April 2024).

### 2.3. Prediction of Gene Duplication Events

The chromosome location of each *FvPAT* was obtained from genome annotations and labeled on chromosomes by OmicStudio tools (www.omicstudio.cn/tool/, accessed on 27 April 2024). The gene duplication events were analyzed by the Multicollinearity Scanning Toolkit (MCScanX, [22]).

### 2.4. Prediction of Protein 3D Structure by AlphaFold

The three-dimensional structure of the conserved DHHC domain was modeled by AlphaFold (https://colab.research.google.com/github/deepmind/alphafold/blob/main/notebooks/AlphaFold.ipynb, accessed on 27 April 2024), and the predicted structure with the highest mean pLDDT (per-residue confidence) of 72.35 (high confidence between 70 and 90) was visualized in PyMOL [23,24].

### 2.5. Analysis of Gene Structure, Conserved Motif, Cis-Regulatory Elements, and Transcription Factors

The introns, exons, and promoter region of 1500 bp upstream from the start codon of each *FvPAT* were obtained from strawberry genome annotations. The conserved motifs were predicted on the online Platform (https://meme-suite.org/, accessed on 27 April 2024) based on MEME algorithm for the maximum expected value (Motif Elicitation, MEME v5.5.0). These elements and motifs were visualized using TBtools-II [25]. The cis-regulatory elements (CREs) of the promoter sequences were analyzed by Plant CARE (http://bioinformatics.psb.ugent.be/webtools/plantcare/html/, accessed on 27 April 2024) and displayed by GSDS 2.0 (https://gsds.gao-lab.org/, accessed on 27 April 2024). The Plant Transcriptional Regulatory Map (PTRM, https://plantregmap.gao-lab.org/, accessed on 27 April 2024) was used to identify potential transcription factor binding sites (TFBS) with a *p*-value ≤ 10^−5^ and the map was generated on hiplot (https://hiplot.com.cn/, accessed on 27 April 2024). The correlation between *FvPATs* and predicted TFBSs was further analyzed and exhibited by Cytoscape software (v3.8.2).

### 2.6. Plant Growth and Phytohormone Treatments

The woodland strawberry seeds were surface-sterilized and placed on solid ½ Murashige and Skoog media (½ MS) containing 1% sucrose. After 48 h stratification at 4 °C, the seeds were germinated, and the seedlings were grown for 15 days under long day (LD) with 16 h light (120 µm m^−2^s^−1^)/8 h dark at 21 °C. The seedlings were transferred to liquid ½ MS without or with ABA (50 µM), SA (100 µM), and IAA (20 µM) and incubated for 6 and 24 h. The seedlings were harvested, frozen in liquid nitrogen, and stored at −80 °C until further analysis.

### 2.7. RNA Isolation and RT-qPCR

Using the RNAPrep Pure plant Plus Kit (Qiagen, Beijing, China), total RNAs were extracted from 15-day-old seedlings, roots, stems, fully expanded leaves, and fully opened flowers of mature plants. Receptacle tissues (fruits), including 4, 14–16, 22–24, 28–30, and 34–36 days after flowering (DAF) were also collected. This was followed by first-strand cDNA synthesis from 500 ng RNAs using the FastKing RT Kit (Qiagen, Beijing, China). For RT-qPCR, a 15-µL reaction with TransStart Top Green qPCR SuperMix (TransGen, Beijing, China) was set up and run on a Rotor-Gene Q RT-qPCR system (Qiagen, Beijing, China). The relative transcript levels were calculated by the 2^−∆∆Ct^ method with the *FvActin* as the internal control and expression in seedlings as 1 [26]. At least three replicates were included in each run. The data were visualized with the Z-score of these genes using the heatmap module in the TBtools-II software. The sequences of all RT-qPCR primers are shown in Supplemental Table S1.

### 2.8. Construction of Yeast Expressing Vectors and Yeast Complementary Assay

The ORF of strawberry *FvPAT19* (FvH4_7g11740), a homolog of Arabidopsis *AtPAT14* [13], was amplified via RT-PCR from total RNAs isolated from leaves. It was cloned in the Gateway vector pDONR-Zeo and recombined to the yeast expression vector pYES-DEST52 that contains a C-terminal V5 epitope tag for protein detection by Western blot (Invitrogen, Loughborough, UK). The construct containing the DHH^C155S^ point mutation was subsequently created using overlapping PCR as described previously [14]. All the primers used for cloning are listed in Table S5.

The yeast transformation and complementation assay were carried out as described before [10]. Images of yeast colonies on plates were scanned. For microscopic observation, cells were observed using phase-contrast light microscopy on Zeiss LSM 710 (Zeiss, Oberkochen, Germany).

### 2.9. Auto-Acylation by the Acyl-mPEG Exchange Assay

For the auto-acylation of FvPAT19 and its point mutant FvPAT19C155S, the acyl-mPEG exchange assay [27] was performed with some modification. Total proteins were extracted from transgenic *akr1* cells expressing FvPAT19-V5 and FvPAT19C155S-V5. After blocking the free thiols with N-Ethylmaleimide (NEM), the protein sample was divided into two equal portions. Hydroxylamine (HA) was added to the HA^+^ sample while HA dissolving buffer was added to the HA^−^ sample. The samples were incubated for 1 h with gentle agitation. The proteins were precipitated and dissolved in a TEA buffer containing 4% SDS and 4 mM EDTA. The mPEG maleimide 10 kD (Sigma, UK) was added and the reaction was incubated at room temperature with gentle agitation for 1 h. The samples were again precipitated, resuspended in 100 μL of 2xSDS sample buffer, and analyzed by SDS-PAGE followed by Western blotting. The detection of FvPAT19 and FvPAT19C155S was carried out with the anti-V5 antibody and ECL method.

## 3. Results

### 3.1. The Woodland Strawberry Genome Encodes 21 Protein S-Acyltransferases

In order to carry out a comprehensive and detailed analysis of protein S-acyltransferases in the woodland strawberry (*F. vesca,* ‘Hawaii 4’), we searched its whole genome protein sequences against the conserved DHHC-CRD (PF01529) commonly found in this family of proteins. The resulting protein sequences were screened for their conserved domains by SMART and NCBI analyzing tools. After eliminating redundant genes, we identified 21 putative S-acyltransferases and named them as FvPAT1-21 according to their locations in the order of chromosome 1 to 7.

**Figure 1:**
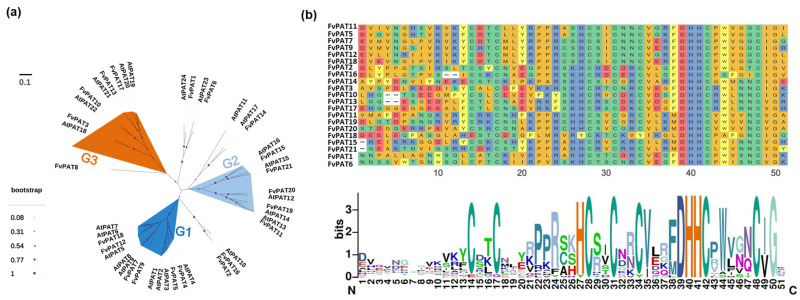
Phylogenetic tree and DHHC domain analysis of the 21 FvPATs from woodland strawberry (*F. vesca*). (**a**) Phylogenetic analysis of FvPATs. The tree is constructed based on the maximum-likelihood method using the protein sequences of FvPATs. AtPATs were used as references. FvPATs and AtPATs were divided into three groups. Group 1 (G1) is shaded darker blue, group 2 (G2) lighter blue, group 3 (G3) orange. (**b**) Analysis of DHHC-CRDs of FvPATs. Top panel, sequence alignment. Same color indicates similarity. Bottom panel, conserved domain display. The larger the font size, the more conserved the amino acid(s) among the FvPATs. Numbers at the bottom indicate amino acid positions.

The protein sequences of the 21 FvPATs were compared with the 24 Arabidopsis AtPATs and a maximum likelihood (ML) phylogenetic tree was constructed, putting these 45 PATs into three main groups. The shortest and the densest branch are the characteristics of group 1 (colored darker blue, Figure 1a). Within this group, FvPAT4, 7, 12, and 18 preserved a high conservation with AtPAT4, 8 and 9, 5, and 6 and 7, while FvPAT5 shares the highest homology to AtPAT3 of Arabidopsis. However, there are no homologous FvPAT(s) to AtPAT1 and 2 in this group. In group 2, each FvPAT is a homolog to one particular AtPAT, such as FvPAT11 to AtPAT13, FvPAT15 to AtPAT16, FvPAT19 to AtPAT14, FvPAT20 to AtPAT12, and FvPAT21 to AtPAT15 (colored light blue, Figure 1a). These one-to-one homologous relationships between strawberry and Arabidopsis PATs can also be observed in group 3, except for FvPAT17 where it shares a high homology of 74% with two AtPATs, AtPAT19 and AtPAT20 (shaded orange, Figure 1a). However, FvPAT2, FvPAT16, and FvPAT14 were not clustered with group 1 or 2, although they were within the same branch. FvPAT8 lacks homology with any AtPATs and was located close to group 3.

The multiple alignment of the protein sequences of all 21 FvPATs showed that they all contain the signature conserved DHHC-CRD domains (Cx2Cx9HCx2Cx4DHHCx5C) [7] with 6 cysteine residues which is similar to the 24 AtPATs in Arabidopsis [20] (Figure 1b, top panel). The further analysis of this domain showed that apart from DHHC at positions 39–42, C^31^ is the most conserved, followed by H^27^C^28^. Additionally, C^48^ and G^50^ near the end of the DHHC-CRD, required for enzyme activity [28], are also conserved in FvPATs; Cys residues at positions 14, 17, and 34 were conserved in some FvPATs. It is also noted that the [R/K]PPR motif at positions 21–24 was present in 11 FvPATs, including FvPAT4, FvPA5, FvPAT7, FvPAT9, FvPAT11, FvPA12, FvPAT15, FvPAT18, FvPAT19, FvPAT20, and FvPAT21 where R24 is the most conserved (Figure 1b, lower panel). This motif is also found in the majority of the Arabidopsis AtPATs, where arginine at the same position is believed to play an important role for enzyme activity [20]. Therefore, within the DHHC-CRD domain, the DHHC and some residues near the end are conserved, while residues at the beginning of this domain are highly variable, which is consistent with AtPATs in Arabidopsis.

PATs identified from other eucaryotes are integral membrane proteins containing four–six predicted TMDs with both the N- and C-termini located in the cytosol [29,30]. This is also true with the 21 FvPATs, where the majority were predicted to possess 4 TMDs apart from FvPAT14 and FvPAT8 which have 5 and 6 TMDs, respectively (Table 1). This, of course, requires further experimentation to verify.

### 3.2. Gene Structure, Chromosome Location, and Prediction of Conserved Motifs of FvPATs

**Figure 2:**
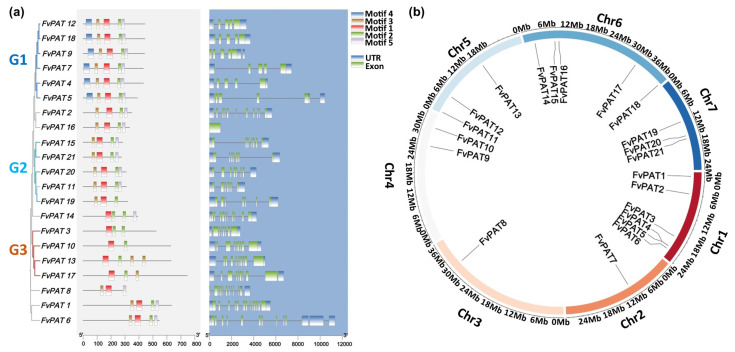
The protein/gene structure and chromosome location of the 21 FvPATs from the woodland strawberry (*Fragaria vesca*). (**a**) Protein and gene structure analysis. G1, G2, and G3 correspond to group 1, 2, and 3 in Figure 1a. Left panel, protein structure (shaded light grey) showing the five conserved motifs. Numbers at the bottom indicate the amino acid number; right panel, gene structure (shaded blue) indicating the UTR and exons. Numbers at the bottom indicate the nuclide acid number. Note that a similar gene structure was found for FvPATs within the same group. (**b**) Chromosome location of the *FvPATs*. The seven chromosomes are represented by different colored bars and their location and size are indicated by the mb on the circle.

The number of nucleotides of the 21 *FvPATs* is considerably variable, ranging from 831 to 2241 and containing 4–13 exons. They encode proteins of 276–746 amino acids with predicted molecular weights (MW) from 31.36–69.26 kDa and pI between 5.68 and 9.61 (Table 1, Figure 2a, left panel). The prediction of the conserved motifs by the MEME algorithm indicates that FvPATs contain five conserved motifs (Figure 2a, left panel). The motifs 1, 2, 3, and 5 (red, green, yellow, and purple) were broadly distributed among most FvPATs, while motif 4 (blue) was only found in the group 1 (G1) FvPATs including FvPAT4, FvPAT5, FvPAT7, FvPAT9, FvPAT12, and FvPAT18, indicating that motif 4 is unique and conserved in the G1 FvPATs. Motifs 1, 2, 3, and 5 were found in group 2 (G2) FvPATs with the exception of FvPAT19 which lacks motif 5. Great variations of these five motifs exist in Group 3 (G3) FvPATs (Figure 2a, left panel). Both 5′ and 3′ UTRs, different numbers of exons and introns (lines) were found in all the *FvPATs* apart from *FvPAT16,* where there was only one large exon present (Figure 2a, right panel). The 21 *FvPATs* were found in all 7 chromosomes with an uneven distribution, where 5 were found on the 1st and 6th, 3 on the 5th and 7th, 2 on the 2nd and 4th, and 1 on the 3rd chromosome, respectively (Table 1, Figure 2b).

### 3.3. One Duplicated Pair of FvPATs, Most FvPATs Synteny with AtPATs

**Figure 3:**
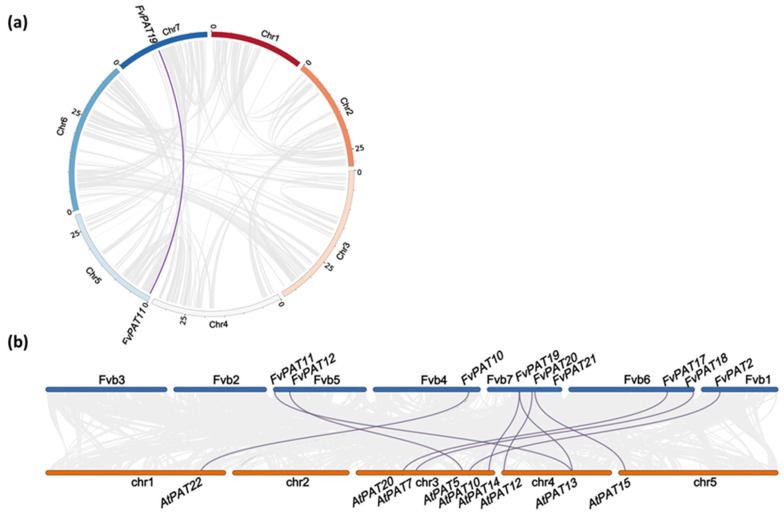
Gene duplication and collinearity analysis of *FvPATs* from woodland strawberry (*F. vesca*). (**a**) Gene duplication events. The seven chromosomes are color-coded and their sizes were indicated by the number of 5 Mbs. The grey lines represent the duplicated fragments in the genome. The duplicated pair of *FvPAT11* and *FvPAT19* are indicated by the purple line. (**b**) Collinearity analysis. The 10 FvPATs that are confirmed to be collinear with AtPATs are joined by purple lines. The woodland strawberry chromosomes (Fv-chr 1–7) are shown on top as thick blue lines and Arabidopsis at the bottom as orange lines (At-chr 1–5). Grey lines in the background indicate the collinear region.

Studies on gene duplication and collinearity between species could shed light on gene evolutionary characteristics and functions. Gene duplication includes whole genome duplication (WGD), and tandem copy and segmental duplication which drives evolution. The analysis of gene duplication events in the woodland strawberry genome shows that there are 118 pairs of duplicated fragments within 1566 pairs of duplicated genes, accounting for approximately 8.39% of all its genes (Table S4). However, only one duplicated pair, i.e., *FvPAT11* and *FvPAT19,* were found (Figure 3a), likely resulting from the gene duplication event (segmental duplication). This agrees with the close phylogenetic relationship and sequence similarity (70.73%) between FvPAT11 and FvPAT19 (Figure 1a).

The number of *PATs* between the woodland strawberry and Arabidopsis is similar, with 21 and 24, respectively. The interspecific collinear analysis between the 24 *AtPATs* and the 21 *FvPATs* reveals that 10 *FvPATs* maintain synteny with *AtPATs*, indicating that they are closely related to each other (Figure 3b).

### 3.4. Regulatory Elements Involved in Hormone and Stress Responses Are Present in the Promoter Regions of FvPATs

**Figure 4:**
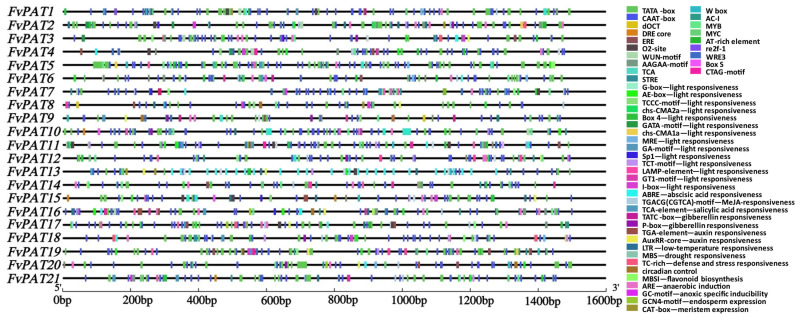
Prediction of the regulatory elements of *FvPATs* of the woodland strawberry (*F. vesca*). The promoter region of 1500 bp upstream of the start codon of each *FvPAT* was analyzed by PlantCARE. CREs, cis-regulatory elements. The positions and different types of the CREs are indicated as different colored boxes and their details are given on the right panel. All CREs predicted were classified into three categories: hormone response elements including ABA, JA, SA, GA, and auxin, the internal regulation element, and basic elements for initiating transcription. The bottom line indicates the 1500 bp of the promotor region.

Cis-regulatory elements (CRE) and transcription factors (TF) are essential for gene expression and function in plant growth, development, and stress responses [31]. Analysis of the promoter region of the 1500 bp upstream of the start codon ATG of each *FvPAT* gene discovered a total of 2371 CREs and 3002 TF binding sites (TFBS), where pro*FvPAT5* possesses the maximum variant of 24 CREs (Figure 4 and Table S2) and pro*FvPAT1* has maximum number of 1505 TFBSs (Table S3). Although, the least number (67) of TFBSs is found in pro*FvPAT8* (Table S3) and pro*FvPAT1* contains the most CRE variants of 27 (Figure 4 and Table S2). The basic elements such as TATA-box and CAAT-box (green and dark-blue boxes, Figure 4) are among the highest number of predicted cis-elements, accounting for 28.7% and 28.5%, respectively (Table S2). Light, hormone, biotic, and abiotic response elements were also present in pro*FvPATs*. For example, we found 14 light-responding elements including G-box, BOX4, TCT-motif, I-box, GT1-motif, MRE, LAMP-motif, AE-box, GATA-motif, and sp1. We also found ABA, JA, SA, GA, and auxin-related elements where JA- and ABA-related elements are among the majority, including ABRE, CGTCA-motif, TGACG-motif, TGA-element, and P-box. Biotic and abiotic stress responses elements, such as the low-temperature element (LTR), drought-inducible element (MBS), defense- and stress-responding element (TC-rich repeats), are also present in the promoter regions of all the 21 *FvPATs*. Additionally, the cell cycle and circadian rhythm control, flavonoid gene synthesis, anaerobic, and hypoxia-specific induction elements are found in *FvPATs*.

**Figure 5:**
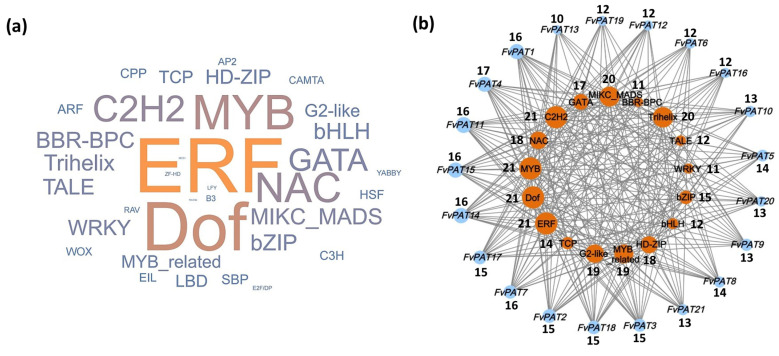
Prediction of transcription factors (TFs) of *FvPATs* of woodland strawberry (*F. vesca*). (**a**) TFs. The font size is positively correlated to the number of TFs. The larger the font size, the higher the number of that TF. Therefore, the order is ERF > Dof > MYB > NAC > C2H2 > GATA > MIKC_MADS > BBR-BPC > Trihelix > TALE. (**b**) Correlation between *FvPATs* and the enriched TFs. Individual *FvPATs* are shown in blue and TFs in red circles. Different sizes of the blue circles indicate different numbers of TFs that each *FvPAT* has, where the larger the circle, the higher the number. Similarly, the size of the red circle positively correlates to the number of each TF. For a particular *FvPAT*, the larger the red circle for a particular TF, the higher this TF is enriched for this *FvPAT*. The number of TFs is indicated next to the circle. Only the top 17 TFs were analyzed.

Given the importance of transcription factors (TFs), we also carried out a detailed analysis for their types. Altogether, we found 3002 TFs belonging to 35 different families for the 21 *FvPATs* (Table S3). The top eleven TFs are ERF (Ethylene Response Factors) (624, 20.8%), Dof (DNA binding with One Finger) (399, 13.3%), MYB (294, 9.8%), NAC (197, 6.6%), C2H2 (182, 6.1%), GATA (119, 4.0%), MIKC_MADS (117, 3.9%), BBR-BPC (111, 3.7%), Trihelix (102, 3.4%), TALE (99, 3.3%), and WRKY (99, 3.3%) (Figure 5a and Table S3). pro*FvPAT4* has the highest number of TFs family of 17, followed by *FvPAT1* (16), *FvPAT7* (16), *FvPAT11* (16), *FvPAT14* (16), and *FvPAT15* (16); the minimal number is 10 of *FvPAT10* when the top 17 TFs were analyzed (Figure 5b). The predominant TF, ERFs, also plant-specific, belong to the subfamily of the AP2/ERF superfamily. As the name indicates, most of the ERFs function downstream of the ethylene signaling pathway, although they are also involved in cytokinin, ethylene, abscisic acid, and other hormone signal transduction pathways [32,33]. The Dof family is a plant-specific TF involved in the regulation of seed germination [34], plant growth and development [35], responses to abiotic stress [36], and multiple hormonal pathways during abiotic stresses [37].

Therefore, the 21 *FvPATs* possess all the regulatory elements involved in hormone and stress responses, and hence, may participate in these biological processes of the woodland strawberry.

### 3.5. FvPATs Are Ubiquitously Expressed with High Transcript Levels Found in Vegetative Tissues and Young Fruits

**Figure 6:**
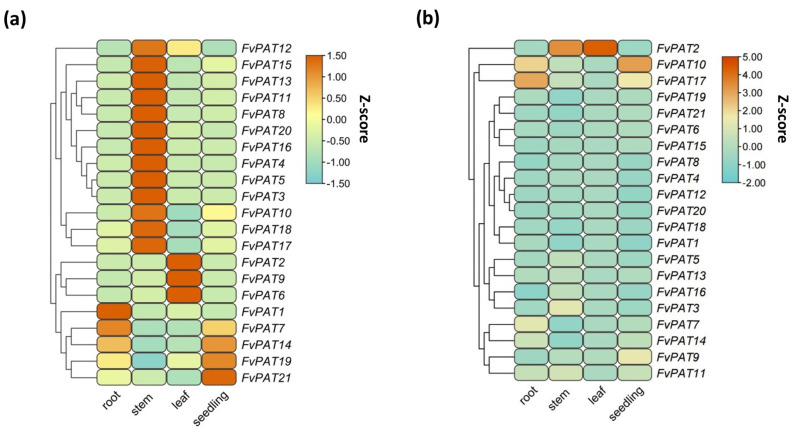
Expression profiles of FvPATs in vegetative of woodland strawberry (*F. vesca*). (**a**) Comparison of expression of individual FvPATs between different tissues. (**b**) Comparison of expression of the 21 *FvPATs* in the same tissue. RT-qPCR was carried out on total RNAs isolated from mature roots, stems, newly expanded leaves, and 14-day-old seedlings. At least three replicates were included in each run. The transcript level was calculated by the 2^−∆∆Ct^ method using *FvActin* as the internal control and the average of the transcript levels of all 21 *FvPATs* in seedlings as 1. The data are visualized using the heatmap module in Tbtools software after being normalized by the Z-score.

We monitored the transcript levels of the 21 *FvPATs* by RT-qPCR from total RNAs isolated from seedlings, different vegetative tissues of mature plants, and different developmental stages of fruits (receptacles). The relative expression values against the house keeping gene *FvActin* were calculated [38]. A ubiquitous expression profile was found for all the 21 *FvPATs,* although a few *FvPATs* showed some tissue preference (Figure 6). For example, *FvPAT1* was highly expressed in the roots with 2–2.5-fold higher than other tissues, *FvPAT2*, *FvPAT6,* and *FvPAT9* in the leaves with 2.5-fold higher than other tissues, while *FvPAT7*, *FvPAT14,* and *FvPAT19* in both the roots and seedlings. The highest expression level of *FvPAT21* was detected in the 15-day-old seedlings, while a similar and low expression was found in the mature vegetative tissues, 2–2.5-fold decreased than that in the seedlings. Interestingly, the stems contained the highest levels of transcripts of the remaining 13 *FvPATs* (Figure 6a). The expression of *FvPAT17* in the roots and *FvPAT10* in the seedlings was at the highest level compared to the other *FvPATs*. In the stems and leaves, the highest transcription was observed with *FvPAT2* among the 21 *FvPATs,* where its expression was 7-fold higher than the other genes in the leaves (Figure 6b).

**Figure 7:**
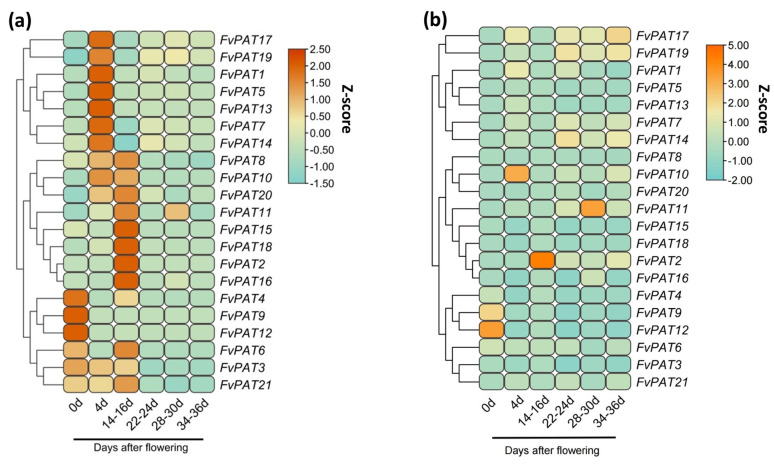
Expression profiles of *FvPATs* in reproductive tissues of woodland strawberry (*F. vesca*). (**a**) Comparison of the expression of individual *FvPATs* in fruits of 0, 4, 14–16, 22–24, 28–30, and 34–36 days after flowering (DAF). (**b**) Comparison of expression of the 21 *FvPATs* in the same aged fruits of 0, 4, 14–16, 22–24, 28–30, and 34–36 DAF. RT-qPCR was carried out on total RNAs isolated from reproductive tissues including the fully opened flower (0 d) and receptacle (fruit) tissues of 4, 14–16, 22–24, 28–30, and 34–36 DAF. At least three replicates were included in each run. The transcript level was calculated by the 2^−∆∆Ct^ method using *FvActin* as the internal control and the average of the transcript levels of all 21 *FvPATs* in fully opened flowers as 1. The data are visualized using the heatmap module in Tbtools software after being normalized by the Z-score.

As strawberry fruit is the organ that holds commercial value, we also monitored the expression profiles of all 21 *FvPATs* during the 6 well-defined fruit (receptacle) developmental stages. This showed that high levels of both *FvPAT3* and *FvPAT21* were found at the early stage between 0–16 days-after-flowering (DAF, Figure 7a). *FvPAT4*, *FvPAT9,* and *FvPAT12* exhibited the highest transcription in the fully opened flowers. However, their expression levels decreased by 3-fold and stayed at the same levels thereafter. *FvPAT8*, *FvPAT10*, and *FvPAT20* reached the highest expression between 4–16 DAF. The expression levels of *FvPAT1, FvPAT5*, *FvPAT7*, *FvPAT13*, *FvPAT14*, *FvPAT17,* and *FvPAT19* increased and peaked at 4 DAF then deceased at 14–16 DAF where *FvPAT1*, *FvPAT5,* and *FvPAT13* decreased by 2.5-fold, and thereafter maintained the same level, while *FvPAT7*, *FvPAT14*, *FvPAT17,* and *FvPAT19* decreased by 2.5–3.5-fold at 14–16 DAF followed by an increase by 0.5–1.5-fold at the 22–24 DAF. The highest expression levels of *FvPAT2*, *FvPAT15*, *FvPAT16,* and *FvPAT18* were detected at 14–16 DAF. However, at the late stage of 28–30 DAF, only the *FvPAT11* transcript showed a 1.5-fold increase, whilst the others stayed at low levels and stable. Among the 21 FvPATs, *FvPAT12* and *FvPAT9* showed the highest expression in the fully opened flower (0 DAF) and about 4–5 fold higher than other *FvPATs* (Figure 7b). In the receptacle (fruit) tissues of 4, 14–16, and 28–30 DAF, the highest transcription was observed with *FvPAT10*, *FvPAT2,* and *FvPAT11*, respectively, 5–6-fold higher than other *FvPATs*.

### 3.6. Changes in Expression Levels of FvPATs in Plate-Grown Seedlings Treated with IAA, ABA, and SA

**Figure 8:**
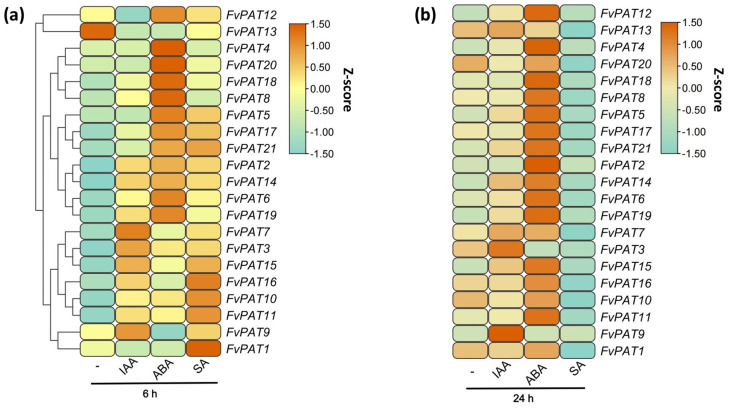
Effect of IAA, ABA, and SA on the Expression profiles of *FvPATs* in the woodland strawberry (*F. vesca*). (**a**) Expression profiles of *FvPATs* treated with IAA, ABA, and SA for 6 h. (**b**) Expression profiles of *FvPATs* treated with IAA, ABA, and SA for 12 h. Seedlings were grown on ½ MS for 14 days and transferred to medium containing 20 µM IAA, 50 µM ABA, and 100 µM SA for 6 and 24 h. At least three replicates were included in each run. The transcript level of each gene was calculated by the 2^−∆∆Ct^ method using *FvActin* as the internal control and the average transcript level of all 21 *FvPATs* in non-treated seedlings at 0 h as 1. The data are visualized using the heatmap module in Tbtools software after being normalized by the Z-score.

Protein S-acyltransferases play important roles in growth and stress responses in Arabidopsis and the other plant species studied so far [13,16,17]. To see if/how the 21 FvPATs function in these processes, we monitored by RT-qPCR the transcript levels of all 21 *FvPATs* in response to IAA to demonstrate growth promoting, ABA, and SA to abiotic and biotic stress response. The relative expression profiles of the 14-day-old plate-grown seedlings were treated with 20 µM IAA, 50 µM ABA, and 100 µM SA for 6 h and 24 h. After 6 h of treatment with IAA, increased expressions for the majority of the *FvPATs* (*FvPAT2, FvPAT3, FvPAT6, FvPAT7, FvPAT8, FvPAT9, FvPAT10, FvPAT11, FvPAT14, FvPAT15, FvPAT16, FvPAT17, FvPAT18, FvPAT19, and FvPAT21*) were detected, where *FvPAT7* showed the highest level of increase by 2.5-fold. However, *FvPAT1, FvPAT12, FvPAT13, FvPAT20* were down-regulated, where *FvPAT13* showed the most reduction by 2.5-fold (Figure 8a). In the 24 h IAA-treated seedlings, a stable 0.5–1.5-fold increase in *FvPAT3, FvPAT6, FvPAT7, FvPAT9, FvPAT11, FvPAT14, FvPAT15, FvPAT19,* and *FvPAT21* was observed compared to the 6 h treated seedlings. The expression of *FvPAT4*, *FvPAT5*, *FvPAT12,* and *FvPAT13* was detected after 24 h of treatment with IAA, although they did not show changes after 6 h of treatment. Notably, *FvPAT1* was the only one with its expression reduced in the 24 h treated samples (Figure 8b). Therefore, most *FvPATs* showed an increased expression in response to IAA.

To see if the expression profiles of *FvPATs* were affected by biotic and abiotic stress, ABA and SA were supplemented in the media. As shown in Figure 8a, after 6 h of treatment by ABA, except *FvPAT1*, *FvPAT9,* and *FvPAT13* being down-regulated, most *FvPATs* were up-regulated, where a 2–2.5-fold increase was also found for *FvPAT2*, *FvPAT4*, *FvPAT5*, *FvPAT6*, *FvPAT8*, *FvPAT14, FvPAT17, FvPAT18*, *FvPAT19,* and FvPAT20, and a 0.5–1.5-fold increase for *FvPAT21, FvPAT3, FvPAT7, FvPAT10, FvPAT11, FvPAT12, FvPAT15,* and *FvPAT16*. Prolonged treatment with ABA for 24 h resulted in more elevated transcript levels for all the *FvPATs,* where *FvPAT2, FvPAT4, FvPAT5, FvPAT12, FvPAT14, FvPAT15, FvPAT19,* and *FvPAT21* were among the most affected (~ 2.5-fold), except for a 1.5- and 0.5-fold decrease for *FvPAT13* and *FvPAT3*, respectively (Figure 8b).

The expression profiles of *FvPATs* in SA-treated seedlings were very different from those in ABA-treated ones, especially in 24 h treated samples. In the 6 h SA-treated seedlings, all, except a 1.5-fold decrease for *FvPAT13* and no change for *FvPAT4* and *FvPAT8,* showed an increased expression, with the maximum 2.5-fold increase found in *FvPAT10* and *FvPAT11* (Figure 8a). However, the expression of most of the *FvPATs* were decreased at least by 1-fold in the 24 h treated seedlings apart from a slight increase for *FvPAT2, FvPAT4, FvPAT5, FvPAT9, FvPAT12, FvPAT15,* and *FvPAT19* compared to the untreated samples (Figure 8b).

Therefore, the combined results clearly demonstrated that the majority of the 21 *FvPATs* responded to IAA, ABA, and SA treatment, implying that S-acylation plays important roles in the phytohormonal, biotic, and abiotic stress responses in the woodland strawberry.

### 3.7. FvPAT19 Is an S-Acyl Transferase

To see if the FvPATs have enzyme activity as protein S-acyltransferases, we carried out a yeast complementation study and S-acylation verification of FvPAT19, because its homolog PAT14 has been well characterized in Arabidopsis, maize, and pear [13,19,39]. FvPAT19 as well as its point mutant FvDHHC^155^S were expressed in the yeast PAT mutant *akr1* strain, which lacks AKR1, one of the seven yeast PATs. This mutant is temperature-sensitive and cannot grow at a high temperature of 37 °C. This growth defect has been used for the verification of enzyme activity of many other PATs from different organisms, including plants before.

**Figure 9:**
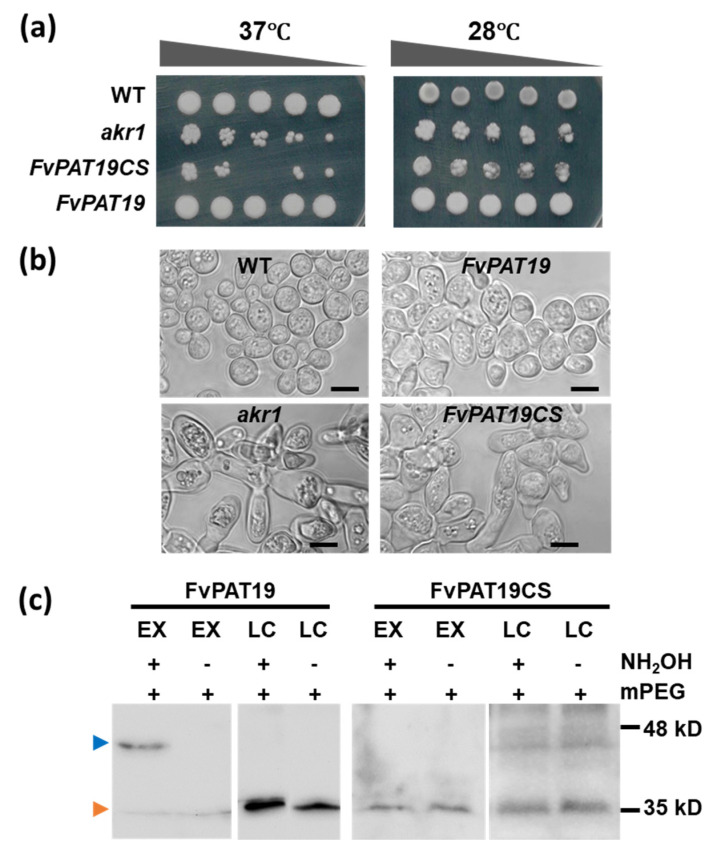
Protein S-acyltransferase 19 of woodland strawberry (*F. vesca*), FvPAT19 has S-acyltransferase activity. (**a**) Yeast growth assay. The wild-type (WT), *akr1*, FvPAT19-, and FvPAT19CS-expressing *akr1* cells were grown at 28 °C and 37 °C for 3 days. The WT yeast grew well but *akr1* did not at 37 °C (**left panel**), although this growth defect was less obvious at 28 °C (**right**). The growth defect of *akr1* at 37 °C was largely restored by expressing FvPAT19 (FvPAT19/*akr1*) but not by FvPAT19CS (FvPAT19CS/*akr1*) in *akr1.* Five microliters of serial dilutions of 1:5, 1:10, 1:20, and 1:40 from 1 OD600 cells were spotted on solid selective medium supplemented with 2% galactose and grown at 28 °C or 37 °C. (**b**) Cell morphology. Cells grown at 37 °C were observed by phase contrast microscopy. The WT cells are individual, small, and round, while the *akr1* cells are large, irregular, and elongated. The *akr1* cells transformed with FvPAT19 largely restored the phenotype with rounder although still larger cells, whilst those transformed with FvPAT19CS resemble the *akr1* cells. The cells were inoculated in liquid selective medium supplemented with 2% galactose and grown at 37 °C for 4 days. Bars = 5 µm. (**c**) Acyl-PEG exchange assays were performed on transgenic *akr1* cells expressing FvPAT19 and FvPAT19CS using 10 kD mPEG-maleimide and analyzed on immunoblots probed with anti-V5 antibody and detected by ECL. The position of PEGylated FvPAT19 is indicated with an asterisk. The molecular weight of FvPAT19 is ~35 kDa. Both FvPAT19 and FvPAT19CS were detected in loading control (LC) with (+) or without (-) NH_2_OH treatment. In the experimental samples (EX), when 10kD mPEG was present, a molecular weight shift by 10 kD for FvPAT19 was detected in the NH_2_OH-treated sample (+), while no such band in the non NH_2_OH-treated sample was present, indicating that FvPAT19 is S-acylated. Therefore, cysteine in DHHC is required for the auto-acylation of FvPAT19.

We first observed the growth of all four genotypes of wild type (WT, positive control), *akr1* (negative control), and transgenic *akr1* expressing either FvPAT19 or FvPAT19^C155S^ at both 28 °C and 37 °C. As shown in Figure 9a, while the WT yeast grew well at both 28 °C and 37 °C, *akr1* did not, showing both the positive and negative controls growth habits as predicted. For the experimental samples, the FvPAT19 expressing transgenic *akr1* showed an appropriate amount of growth at 37 °C, although still less than WT, whilst transgenic *akr1* expressing FvPAT19^C155S^ failed to grow at 37 °C (Figure 9a). Further observation and comparison of the cell morphology of these four genotypes were carried out (Figure 9b). This showed that, while the WT yeast cells were typically round, small at 13.4 ± 3.59 µm^2^, and well dispersed, about 60% of the *akr1* cells were elongated and clumped together, which were double the size of WT at approximately 26.8 ± 5.49 µm^2^. Interestingly, the shape and size of the FvPAT19-expressing *akr1* cells exhibited a different phenotype which were rounder than *akr1* but longer than WT, while they were smaller than *akr1* but still much larger than WT by about 1.5-fold. Some of these cells were still clumped together, although the proportion was reduced to ~23.5%. By contrast, the FvPAT14C^155^S-expressing *akr1* cells were indistinguishable from *akr1* (Figure 9b). Therefore, FvPAT19 can partially rescue the temperature-sensitive growth defect of *akr1* mutant, while their point mutant cannot.

To see if FvPAT19 is auto-acylated, the acyl-mPEGyl exchange gel shift assay was carried out [27]. For this, the unmodified cysteine thiol groups in FvPAT19 yeast cell lysates were first blocked by the sulfhydryl reactive reagent NEM. They were then treated with the S-acyl group cleavage reagent hydroxylamine (+NH_2_OH) to release thioester-linked S-acyl moieties, restoring the modified cysteine to thiols (-SH), which were then PEGylated with the maleimide group. The PEGylated proteins can be detected by Western blotting to monitor the molecular weight increase by 10 kD. In the negative control (-NH_2_OH), no free sulfhydryls were generated; S-acylated proteins do not react with mPEG-Mal and, hence, are not detected. Figure 9c shows that FvPAT19-V5 at ~35.6 kDa was detected in both loading controls (LC) (orange arrowhead). For the FvPAT19-V5-mPEG-Mal at ~45.6 kDa, a shift by 10 kD was also detected in the NH_2_OH-treated sample (blue arrowhead), while no band shift was detected in the -NH_2_OH sample (left panel, Figure 9c). Similarly, FvPAT19CS was captured in LC samples, but no MW shift was detected for FvPAT19CS (right panel, Figure 9c). Therefore, FvPAT19 is auto-S-acylated and cysteine within DHHC is required for its S-acylation activity.

### 3.8. 3D Structure Prediction of FvPAT19, Its DHHC and DHHC^155^S

**Figure 10:**
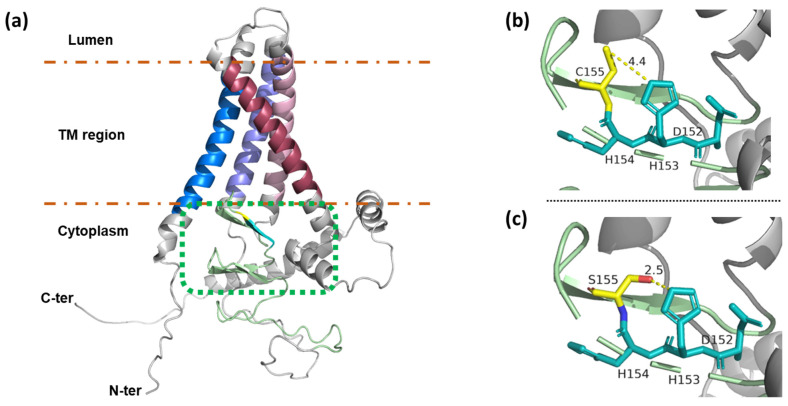
3D structure prediction of FvPAT19, its DHHC and DHHC155S. (**a**) The 3D protein structure of FvPAT19 predicted by AlphaFold 2. The 4 TMs as α-helices (blue, purple, pink, and raspberry-red) were embedded between the lipid bilayers (brown dotted lines) of the membrane, while the N- and C-termini are in cytosol. The DHHC-CRD domain (green dotted box) is also in cytosolic, where DHHC is in the proximity of the membrane. (**b**) 3D structure of DHHC motif. The length between D^152^- and C^155^ is 4.4 (yellow dotted line) supporting C^155^ binding to the fatty acyl chain. D^152^, H^153^, and H^154^ (colored cyan), and C^155^ (colored yellow) are indicated. (**c**) 3D structure of DHHS. The length between D^152^ and S^155^ was reduced to approx. 2.5 (yellow dotted line) due to the formation of a hydrogen bond with H^153^ after the mutation from cysteine (-SH) to serine (-OH). The oxygen atom is colored red.

We constructed the 3D structures of FvPAT19, its DHHC and DHHC155S motifs using AlphaFold 2. As shown in Figure 10a, the four transmembrane (TM) helices formed a teepee-like structure between the phospholipid bilayer with both N- and C-termini in the cytoplasm, where its C-terminus could interact extensively with the DHHC-CRD (shaded green). The two short loops (grey line) that link TM1 (blue), TM2 (purple), TM3 (pink), and TM4 (raspberry-red) are on the luminal side of the membrane, while the highly conserved DHHC-CRD between TM2 and TM3 is embedded inside the cavity surrounded by the four TM helices, where the fatty acyl chain is supposed to bind when S-acylation occurs [40]. The length between D^152^ and C^155^ within the DHHC motif is predicted to be 4.4 Å, supporting C^155^ binding to the fatty acyl chain (Figure 10b, yellow dotted line). However, when C^155^ is mutated to serine, the -SH is replaced by -OH (the oxygen atom is colored red, Figure 10c). This results in the reduced length between D^152^ and S^155^ to 2.5 Å (Figure 10c, yellow dotted line) due to the formation of a hydrogen bond with H^153^, restricting free thiol binding with the fatty acyl chain; hence, no palmitoylation could occur.

## 4. Discussion

We have identified 21 DHHC-CRD-containing putative PAT protein sequences from the woodland strawberry genome. This number is comparable to the 24 PATs isolated from Arabidopsis [20]. Different numbers of PATs ranging from 6 (*Volvox carteri*) to 52 (*Panicum virgatum*) are present in the 31 different plant genomes studied [17]. The increased number of genes is often caused by gene duplication events during evolution. In the case of the woodland strawberry, we found that one pair of genes, *FvPAT11* and *FvPAT19,* are the product of gene duplication (Figure 3a). This is consistent with Arabidopsis, where AtPAT13 and AtPAT14 are closely related and play additive roles in SA-mediated premature leaf senescence [12,13]. Therefore, FvPAT11 and FvPAT19 could play roles in the woodland strawberry similar to AtPAT13 and AtPAT14 in Arabidopsis, given their similar sequence [12,13] (Figure 1a). A previous survey of the apple genome, another species in the Rosaceae family, identified a much large number of 33 PATs. This expansion compared to 21 in the woodland strawberry is likely caused by tandem and segmental duplications as well as whole genome duplications found with the apple genome [18]. According to the protein sequence similarity, the 21 FvPATs and the 24 AtPATs are categorized into 3 main groups with exceptions for AtPAT10/FvPAT2 and 16, AtPAT17/FvPAT14, AtPAT23/FvPAT6, and AtPAT24/FvPAT1 which do not fall into any of the 3 groups (Figure 1a). There is also an orphan FvPAT, FvPAT8, that does not share a high homology to any of the 24 AtPATs, although it is close to group 3 (Figure 1a), as well as containing a conserved DHHC-CRD (Figure 1b). Otherwise, we can find all other corresponding AtPAT homologs in strawberries, despite the reduced number and lower redundancy in FvPATs. For example, there is only one FvPAT, FvPAT5, for three AtPATs, AtPAT1, AtPAT2, and AtPAT3; one FvPAT18 for AtPAT6 and AtPAT7 within group 1; and one FvPAT17 for AtPAT19 and AtPAT20 within group 3 (Figure 1a). Therefore, the *PATs* are less diverged in the woodland strawberry than in Arabidopsis.

All the 21 FvPATs have the conserved DHHC-CRD, the signature motif for this family of proteins found in all the characterized S-acyltransferases so far (Figure 1b). All the Cys residues within the DHHC-CRD are highly conserved between FvPATs in the woodland strawberry and AtPATs in Arabidopsis (Figure 1b) [20]. Another motif, [R/K]PPR, related to enzyme activity was also found in some FvPATs, which is consistent with AtPATs where the last R is highly conserved (Figure 1b) [20,41]. Most FvPATs are predicted to have four TMDs, except FvPAT14 and FvPAT8, which are predicted to have five and six TMDs, respectively (Table 1). However, this remains to be determined given that their homologous AtPAT17 in Arabidopsis was predicted to have six TMDs [20]. The odd number of five TMDs in FvPAT14 may be due to incorrect annotation. Since the genome of *Fragaria vesca* was first sequenced and assembled in 2011, there have been several updates, with the most recent one in 2019 [42,43], which was the version we used for identifying FvPATs in this study. While this version likely represents an improvement in annotation accuracy, it is still a “work in progress.” Until a more refined genome assembly of the woodland strawberry becomes available, the predicted number of TMDs in FvPAT14 cannot be definitively determined. Nevertheless, this does not change the fact that FvPATs possess features typical of PATs and may function similarly to AtPATs in Arabidopsis.

It was reported that the expression of human DHHC-1, 3–10, 12–14, 16–18, and 20–22 are detected widely in different tissues while DHHC-11 was only expressed in testis [30]. DHHC proteins in Drosophila supports these tissue-specific expression patterns, such as CG4483, CG4956, CG13029, CG17075, CG17195–17198, CG17287, and CG18810 only expressed in testis [44]. Expression profiles of the 24 AtPATs in Arabidopsis showed that they are ubiquitously expressed, suggesting that they play roles throughout the life cycle of Arabidopsis [20]. We also analyzed the expression profiles by RT-qPCR using total RNA isolated from seedlings, and vegetive and reproduction tissues. Our results showed that the 21 *FvPATs* were expressed ubiquitously in woodland strawberry although the levels are different between different *FvPATs* in the different tissues tested (Figure 6). *FvPAT21* showed the highest expression level in seedlings than any other vegetative organs (Figure 6a), indicating that it may be involved in seedling establishment and growth. In vegetative organs, the higher transcript of *FvPATs* was *FvPAT2*, *FvPAT10,* and *FvPAT17* compared to other *FvPATs* (Figure 6b). It is highly likely that these three genes are involved in the growth of vegetative organs.

In Arabidopsis, the specific expression of some PATs was found in reproductive organs where increased transcript levels of *AtPAT1, 2,* and *3* were found during flower development, where *AtPAT2* and *AtPAT3* showed a high expression in stamen and pollen. Floral-specific expression was also found with *AtPAT10* and *AtPAT21* [20]. In line with these, much-reduced seed production was found in the *atpat10* caused by defects in pollen tubes, whilst no seeds were produced in *atpat21* due to defects in both male and female gametogenesis [10,15]. Therefore, this clearly demonstrates that S-acylation plays essential roles in the reproduction of Arabidopsis. These data are particularly relevant to strawberries given that the fruits are the marketing organ. Therefore, we monitored the expression of all 21 *FvPATs* during fruit development. The highest transcript levels were detected for the majority of the 21 *FvPATs* during the early stages (0–16 DAF) of fruit growth and development, although the timing to reach and maintain the maximum expression were different among these genes (Figure 7), indicating that S-acylation mediated by FvPATs is pivotal for strawberry fruit development.

S-acylation is involved not only in growth and development, but also in biotic and abiotic stress signaling pathways [45]. For example, AtPAT10 participates in the salt response, via targeting the calcium sensors CBL2 and CBL3 to tonoplast [46]. Through S-acylating and targeting P2K1 to the plasma membrane, AtPAT5 and AtPAT9 regulate immunity in Arabidopsis [47]. In this study, we monitored the changes in transcript levels in strawberry seedlings upon treatment with the growth promoting hormone auxin IAA, and the two growth inhibiting hormones, ABA, the abiotic, and SA, the biotic stress signals, respectively. Consistent to the increased expression levels for the majority of the *DHHC* genes in rice when treated with IAA [16], nearly half of the *FvPATs* were also up-regulated within the first 6 h of IAA treatment, indicating these *FvPATs* may play positive roles in the IAA signal pathway (Figure 8a). Also, in agreement with rice, *DHHCs* is the treatment with ABA and SA where the rapid elevation in transcript levels of most *FvPATs* is also detected (Figure 8a). In Arabidopsis, AtPAT13- and AtPAT16-mediated S-acylation of R5L1 is crucial for its PM targeting to activate the plant defense response [48]. Notably, both the homologous genes *FvPAT11* and *FvPAT15* were also rapidly up-regulated by SA, implying that they may also function in disease resistance in strawberry via similar mechanisms.

The RT-qPCR data are largely in agreement with the analysis of cis-elements and TF biding sites of the promotor regions of the 21 *FvPATs,* where three main cis-elements were identified, i.e., (1) hormone response elements including ABA, JA, SA, GA, and auxin; (2) the internal regulation elements consisting of cell cycle and circadian rhythm control, flavonoid gene synthesis, and anaerobic and hypoxia-specific induction; and (3) basic elements for initiating transcription (Figure 4 and Figure 5, Tables S2 and S3).

To see if the 21 FvPATs function as S-acyltransferases, we studied the enzyme activity of one of them, FvPAT19 by complementation and auto-acylation assays in yeast. This confirmed that FvPAT19, like its homolog AtPAT14 in Arabidopsis, functions as a PAT and its enzyme activity requires the Cys within DHHC (Figure 9c). The 3D protein structure prediction by Alphafold 2 was in agreement with this result (Figure 10). This structure prediction is also in agreement to the first atomic structure of hDHHC20 determined using X-ray crystallography [40].

## 5. Conclusions

In summary, we identified 21 DHHC-CRD-containing protein sequences from the woodland strawberry genome, FvPAT1-21. A bioinformatic analysis showed that they are typical for this family of proteins. While most of the FvPATs have a broad expression profile, many of them were expressed in high levels during the early stages of fruit development, suggesting that S-acylation is involved in the fruit development of strawberries. The upregulation of transcript levels of the majority of the *FvPATs* in seedlings treated with IAA, ABA, and SA supports the notion that FvPATs-mediated S-acylation is required for growth, development, and the response to abiotic and biotic stress. The data reported here could contribute to the further dissection and understanding of the biological function and mechanisms of S-acylation mediated by the PAT family in strawberries.

## Figures and Tables

**Table 1:** List of the 21 FvPATs of the woodland strawberry and their important characteristics.

Name	Gene ID	Chr.	AAs	MW (KDa)	pI	Instab. Index	A.I.	GRAVY	TMDs	N in Cytosol
FvPAT1	FvH4_1g02030	Chr1	634	69.2	6.29	29.4	84.1	−0.21	4	Yes
FvPAT2	FvH4_1g10780	Chr1	348	39.6	8.95	44.2	96.6	0.15	4	Yes
FvPAT3	FvH4_1g26210	Chr1	524	59.5	9.61	49.6	86.0	−0.17	4	Yes
FvPAT4	FvH4_1g28800	Chr1	432	49.3	5.68	31.3	85.7	−0.16	4	Yes
FvPAT5	FvH4_1g29200	Chr1	389	44.3	8.85	33.0	85.8	−0.08	4	Yes
FvPAT6	FvH4_2g10900	Chr2	546	60.1	6.27	35.3	90.1	−0.04	4	Yes
FvPAT7	FvH4_2g13200	Chr2	431	48.7	6.64	42.0	85.2	−0.15	4	Yes
FvPAT8	FvH4_3g35813	Chr3	307	34.6	8.88	46.1	110.8	0.41	6	Yes
FvPAT9	FvH4_4g22230	Chr4	440	49.6	7.81	41.1	76.1	−0.15	4	Yes
FvPAT10	FvH4_4g30660	Chr4	628	68.8	9.05	57.1	80.8	−0.18	4	Yes
FvPAT11	FvH4_5g00750	Chr5	310	35.1	7.09	45.5	106.0	0.43	4	Yes
FvPAT12	FvH4_5g08980	Chr5	443	49.1	7.92	41.6	80.1	−0.13	4	Yes
FvPAT13	FvH4_5g25830	Chr5	630	69.3	8.35	60.6	72.4	−0.34	4	Yes
FvPAT14	FvH4_6g04920	Chr6	393	44.8	8.83	30.8	101.9	0.26	5	No
FvPAT15	FvH4_6g13410	Chr6	284	32.0	7.97	50.1	95.4	0.24	4	Yes
FvPAT16	FvH4_6g14950	Chr6	333	37.8	8.59	39.0	97.7	0.24	4	Yes
FvPAT17	FvH4_6g39520	Chr6	746	80.4	8.38	53.1	75.2	−0.22	4	Yes
FvPAT18	FvH4_6g53970	Chr6	443	49.8	8.21	37.5	75.6	−0.22	4	Yes
FvPAT19	FvH4_7g11740	Chr7	319	35.6	9.32	34.4	107.0	0.40	4	Yes
FvPAT20	FvH4_7g17410	Chr7	308	34.8	7.11	40.3	99.0	0.34	4	Yes
FvPAT21	FvH4_7g18860	Chr7	276	31.6	8.78	36.3	92.8	0.26	4	Yes

MW, molecular weight; pI, isoelectric point; A.I, aliphatic index; GRAVY, grand average of hydropathicity score; and N in Cytosol, protein N-terminus ends to cytosol.

## Data Availability

The original contributions presented in this study are included in the article/supplementary material. Further inquiries can be directed to the corresponding author.

## Supplementary Materials

The following supporting information can be downloaded at https://www.mdpi.com/article/10.3390/plants14010127/s1, Table S1: qRT-PCR primers of *FvPATs*. Table S2: CREs analysis of the 21 FvPATs identified in this study. Table S3: TFs analysis of the 21 FvPATs identified in this study. Tale S4: 118 replicated fragments identified in this study. Table S5: cloning primers of *FvPAT19*.
